# Genome-Wide Identification and Expression Analysis of Phytosulfokine Peptide Hormone Genes in *Camellia sinensis*

**DOI:** 10.3390/ijms26062418

**Published:** 2025-03-07

**Authors:** Fengshui Yang, Lan Zhang, Qiuying Lu, Qianying Wang, Yanjun Zhou, Qiuhong Wang, Liping Zhang, Kai Shi, Shibei Ge, Xin Li

**Affiliations:** 1 Key Laboratory of Tea Quality and Safety Control, Ministry of Agriculture and Rural Affairs, Tea Research Institute, Chinese Academy of Agricultural Sciences, Hangzhou 310008, China; fengshuiy@163.com (F.Y.); zhanglan@tricaas.com (L.Z.);; 2 National Key Laboratory for Tea Plant Germplasm Innovation and Resource Utilization, 230036 Hefei, China; 3 Department of Horticulture, Zhejiang University, Hangzhou 310018, China

**Keywords:** phytosulfokine, *Camellia sinensis*, genome-wide analysis, gene expression, stress response

## Abstract

Phytosulfokine (PSK) is a tyrosine-sulfated pentapeptide found throughout the plant kingdom, playing key roles in plant growth, development, and responses to biotic and abiotic stresses. However, there is still a lack of a comprehensive analysis of the *CsPSK* gene family in *Camellia sinensis*. In this study, we conducted a genome-wide identification and characterized 14 *CsPSK* genes in tea plants, which are unevenly distributed across seven chromosomes. *CsPSK* genes encode proteins ranging from 75 to 124 amino acids in length, all belonging to the PSK-α type and containing conserved PSK domains. A synteny analysis revealed that the expansion of the *CsPSK* gene family is primarily attributed to whole-genome duplication, with homology to *Arabidopsis thaliana PSK* genes. A promoter region analysis identified cis-regulatory elements related to hormone and stress responses. An expression profile analysis showed that *CsPSK* genes are highly expressed in roots, stems, flowers, and leaves, and are induced by both biotic and abiotic stresses. Furthermore, an RT-qPCR assay demonstrated that the expression levels of *CsPSK8*, *CsPSK9*, and *CsPSK10* are significantly upregulated following *Discula theae-sinensis* infection. These findings establish a basis for further research into the role of the Cs*PSK* gene family in tea plant disease resistance and underlying molecular mechanisms, offering valuable perspectives for developing novel antimicrobial peptides.

## 1. Introduction

Polypeptides, ranging from several to hundreds of amino acids, play an essential role in regulating plant growth, development, and resilience to numerous stress conditions [1,2]. As crucial bioactive small molecules, they mediate cell-to-cell communication through interactions with their receptors [1]. Thus far, extensive genomic analyses have led to the discovery of a wide variety of sulfated peptides, and the potential functions of many of these peptides have been elucidated [3,4,5]. Phytosulfokine (PSK) is a critical plant peptide hormone that plays a central role in regulating diverse processes, including plant growth, developmental pathways, and immune responses [6,7]. Phytosulfokine was the first sulfated peptide identified in plants, reported in *Asparagus officinalis* [5,8]. PSK peptides are generally derived from 75 to 123 amino acid precursors, which is ubiquitous in plants [9]. The *PSK* gene family can be divided into PSK-α (Y_SO3H_IY_SO3H_TQ), -γ (Y_SO3H_VY_SO3H_TQ), -δ (Y_SO3H_IY_SO3H_TN), and -ε (Y_SO3H_VY_SO3H_TN), based on polypeptide sequences [7,9]. Extensive experiments have shown that PSK signaling is involved in plant immune response. Silencing PSK signaling genes increases tomato susceptibility to the fungal pathogen *Botrytis cinerea*, and PSK-induced immune responses depend on the detection of PSKs by the receptor PSKR1 [10]. PSK signaling mediated by PSKR1 diminishes the immune response to *Pseudomonas syringae*, but application of exogenous PSK promoted plant growth in *Arabidopsis thaliana* [11]. Furthermore, PSK signaling has been shown to enhance susceptibility to downy mildew in *A. thaliana* [12]; however, overexpression of the PSK genes *AtPSK2*, *AtPSK4*, and *AtPSKR1* in *A. thaliana* markedly improves resistance against pathogens, highlighting the distinct roles of PSK in plant immune responses [13]. Additionally, PSK stimulates mitotic events for cell proliferation [8], enhances root elongation and numbers in *Medicago truncatula* [14], promotes fruit ripening and quality in tomato [15], and improves drought stress tolerance in *A. thaliana* [16].

Tea (*Camellia sinensis* L.) is one of the major cash crops for producing non-alcoholic beverages [17]. The optimal growing conditions for tea plants are generally warm and humid, which also facilitates pathogen incidence. Anthracnose, a ubiquitous and serious disease, negatively impacts the growth of tea plants and diminishes the quality of tea products [18]. The identification of the causal pathogen of anthracnose in Chinese tea plants (*C. sinensis*) remains controversial. Some researchers propose that *Discula theae-sinensis* is one of the predominant species that causes anthracnose in *C. sinensis*, leading to disease lesions on tea leaves [19,20]. Although PSK has been implicated in the resistance against various plant pathogens [10,11,21,22,23], the characteristics of the *PSK* gene family have not been systematically analyzed in tea plants and little attention has been given to the role of *CsPSKs* in disease resistance.

To date, the PSK gene family has been identified with eight members in tomato [10], seven in Arabidopsis [5], sixteen in *Glycine max* [24], and five in *Lotus japonicus* [25]. In the present study, 14 *CsPSK* genes were first obtained based on the tea plant genome database. We conducted a genome-wide identification of PSK gene family members and analyzed their physicochemical properties, chromosomal traits, phylogenetic relationships, gene conservation, and cis-acting elements to explore the evolution and characteristics of the *CsPSK* gene family in *C. sinensis*. In addition, the expression patterns and stress responses of the *CsPSK* gene family were validated by RNAseq data and RT-qPCR in *C. sinensis*. This study lays the foundation for elucidating the function of *CsPSK* peptides and their potential future applications in *C. sinensis*.

## 2. Results

### 2.1. Genome-Wide Identification and Chromosome Location of the PSK Gene Family in Tea Plants

A total of 14 *CsPSK* gene family members have been identified based on the *C. sinensis* genome and named *CsPSK1* to *CsPSK14* according to the Gene ID genome (Table 1). The length of amino acids in these PSKs is similar, and their size ranges from 75 to 124. The MW (molecular weight) distribution of CsPSK protein is between 8.40 and 14.06 kDa, and the theoretical pI values range from 4.77 to 9.51, among which 3 CsPSK proteins have a pI value greater than 7.0. The hydrophilicity of CsPSK proteins ranges from −0.352 to −0.067. The CsPSK proteins are primarily localized in the nucleus, while CsPSK7, CsPSK11, and CsPSK12 are mainly found in the chloroplast. Interestingly, CsPSK3, CsPSK5, and CsPSK10 are distributed in both the nucleus and chloroplast.

**Figure 1:**
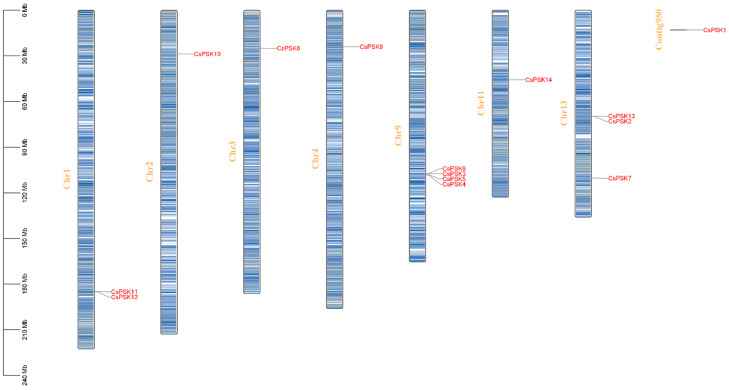
Chromosomal localization of the *CsPSK* gene family members in tea plants. Chromosome numbers are labeled on the left in organe font color (abbreviated as Chr), while gene positions are indicated on the right in red font color.

The 14 *CsPSK* genes are unevenly distributed on seven chromosomes (Chr01, Chr02, Chr03, Chr04, Chr09, Chr11, Chr13) of *C. sinensis* (Figure 1). Among these chromosomes, Chr09 has the highest number of *CsPSK* genes with four members, including *CsPSK3*, *CsPSK4*, *CsPSK5* and *CsPSK6*. *CsPSK10*, *CsPSK8*, *CsPSK9* and *CsPSK14* are found on Chr02, Chr03, Chr04, and Chr11, respectively. *CsPSK2*, *CsPSK7*, and *CsPSK13* are found on Chr13, and *CsPSK11* and *CsPSK12* are identified on Chr01. Furthermore, the *CsPSK1* gene was mapped to Contig950 but remained unanchored on chromosomes.

### 2.2. Phylogenetic Relationship and Synteny Analysis of CsPSKs

**Figure 2:**
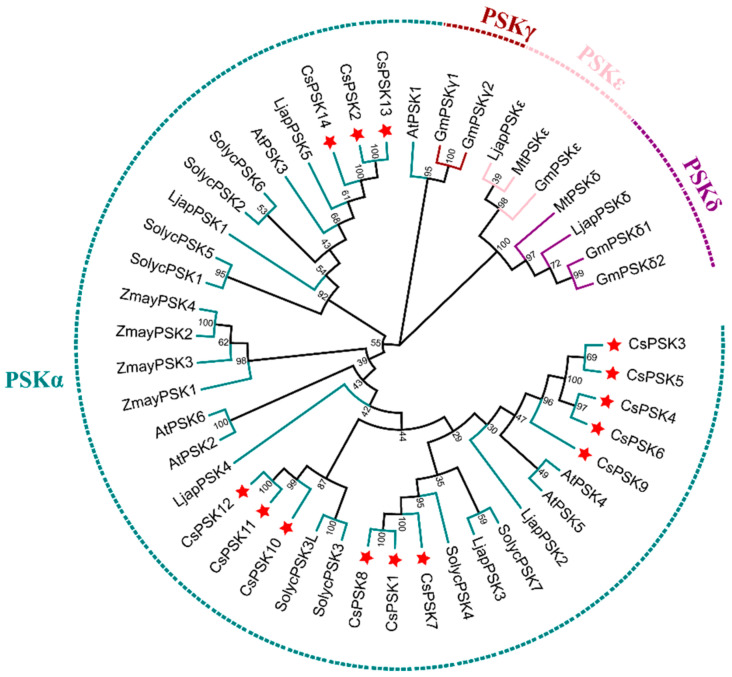
Phylogenetic relationships of the *CsPSK* gene family in *C. sinensis* and other plant species. The sequences of PSKs used in this analysis are provided in Table S1. Red pentagrams indicate CsPSK proteins. Different clades are highlighted in distinct colors.

To elucidate the evolutionary relationship of *CsPSKs* with other homologous genes, a phylogenetic tree has been constructed using previously reported amino acids, including monocot and dicot plant species (Figure 2). The results showed that the *PSK* gene family can be divided into four parts, including PSKα clade, PSKγ clade, PSKε clade, and PSKδ clade. Among them, all the *CsPSKs* are classed into PSKα clade and can be broadly categorized into three branches, indicating that these genes exhibit high homology and share an identical pentapeptide sequence. The CsPSKs of tea plants are mainly clustered with those of Arabidopsis, *Lotus japonicus*, and tomato, such as CsPSK10, CsPSK11, and CsPSK12, and they are grouped within the same clade as SolycPSK3 and SolycPSK3L. The CsPSK2, CsPSK13, and CsPSK14 are located in different subclades from the other CsPSKs. Interestingly, the PSK genes in monocot maize are primarily clustered within the PSKα clade, forming a distinct subclade. Furthermore, the currently known PSKγ, PSKε, and PSKδ genes are predominantly found in leguminous plants. The pentapeptides of PSKγ, PSKε, and PSKδ differ from that of PSKα, suggesting that they may have distinct physiological functions.

**Figure 3:**
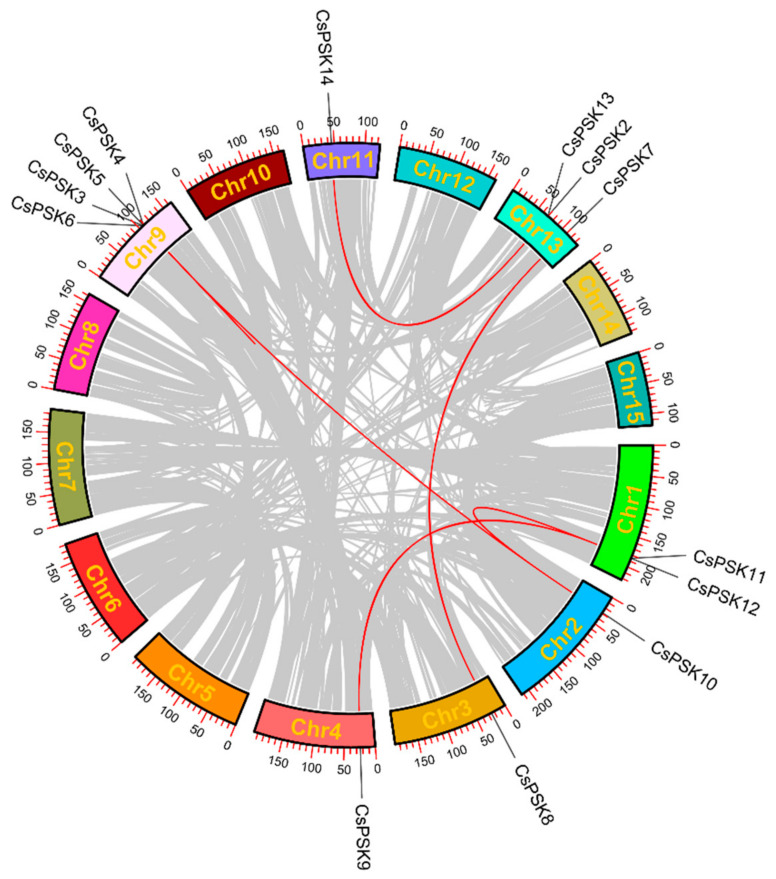
Synteny analysis of *CsPSK* genes in *C. sinensis*. Red lines represent duplicated gene pairs, while gray lines indicate syntenic gene pairs in the whole genome.

To further illuminate the evolutionary significance of the *CsPSK* gene family, we conducted a synteny analysis to investigate events of gene duplication and amplification. There are 10 WGD (Whole Genome Duplication)/segmental duplications, two tandem duplications, one dispersed duplication, and one proximal duplication in the *CsPSK* gene family (Table S2). Several *CsPSKs* have experienced more than one duplication event. The collinearity analysis of seven pairs of *CsPSKs* were identified to participate in the large segmental duplication and evolution of tea plants (Figure 3). Furthermore, we calculated the Ka/Ks ratios for these *CsPSK* gene pairs to examine the impact of selective pressure on gene evolution (Table S3). The results indicated that the Ka/Ks ratios of seven gene pairs were <1.0, suggesting that these genes may have experienced purifying selection during evolution.

**Figure 4:**
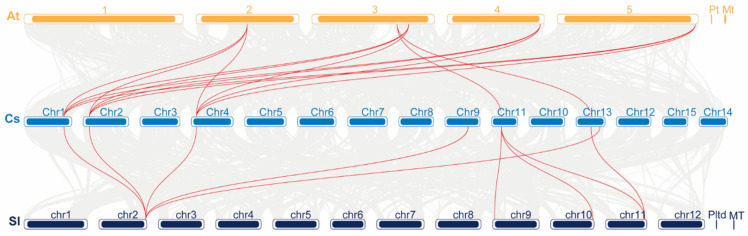
Synteny analysis of *PSK* genes among *C. sinensis*, *A. thaliana, S. lycopersicum*. Cs represents the tea plant genome (sky blue), At represents the Arabidopsis genome (soft amber), and Sl represents the tomato genome (deep blue). Gray lines represent syntenic relationships among different genomes and red lines indicate syntenic relationships among the *PSK* genes.

Simultaneously, a synteny analysis was conducted among *C. sinensis*, *A. thaliana*, and *S. lycopersicum* to elucidate the evolution of the *CsPSK* gene family. The results showed that *C. sinensis* had 14 synteny *PSK* genes with *A. thaliana*, while 10 synteny *PSK* genes were obtained between tea plant and tomato (Figure 4 and Table S4). Several *CsPSK* genes were found as individual homologs between *AtPSKs* and SlPSKs. No homologous *PSK* genes with *A. thaliana* were detected on chromosomes 3 and 9 of *C. sinensis*, while a gene on tomato chromosome 2 shows collinearity with chromosome 9 of the tea plant, indicating that they may have different evolutionary relationships. In addition, multiple single genes in both *S. lycopersicum* and *A. thaliana* show collinearity with several genes in the tea plant, suggesting that functional differentiation may have occurred during evolution.

### 2.3. Sequence Conservation and Gene Structure Analyses of CsPSKs

**Figure 5:**
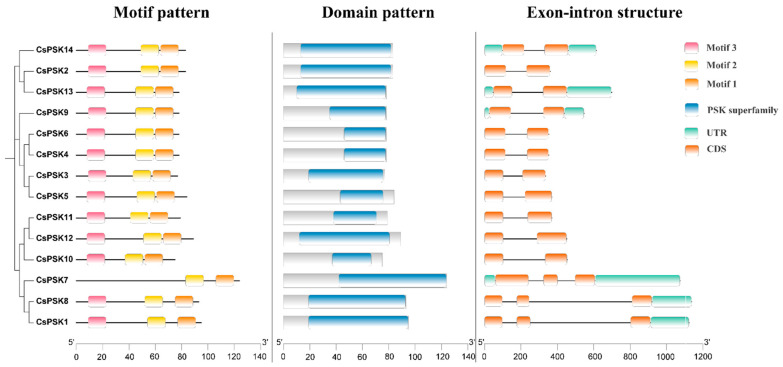
The phylogenetic tree, conserved motif, domain and gene structure of the CsPSK proteins. Different motif patterns are indicated by different colored numbered boxes. The blue squares represent the PSK superfamily in the domain pattern. The distribution of untranslated regions (UTRs) and coding sequences (CDSs) of the *CsPSK* gene family members. The soft green gradient represents UTRs and gradual orange represents CDSs.

Next, we investigated the gene sequence and structure of *CsPSKs* to gain a clearer understanding of conserved structure, physiological function, and evolutionary divergence. A conserved motif analysis reveals that 13 *CsPSK*s contain three motifs, while *CsPSK7* has two motifs, and most of them have the same order (Figure 5). Each of the *CsPSK* genes contained the PSK superfamily domain and exhibited strong conservation. We also analyzed the exon–intron structures within the *CsPSK* gene family. The result showed that the exon members are from 2 to 3 in the *CsPSK* gene family. Most *CsPSKs* in the same group exhibit similar patterns of exon–intron distribution and position.

**Figure 6:**
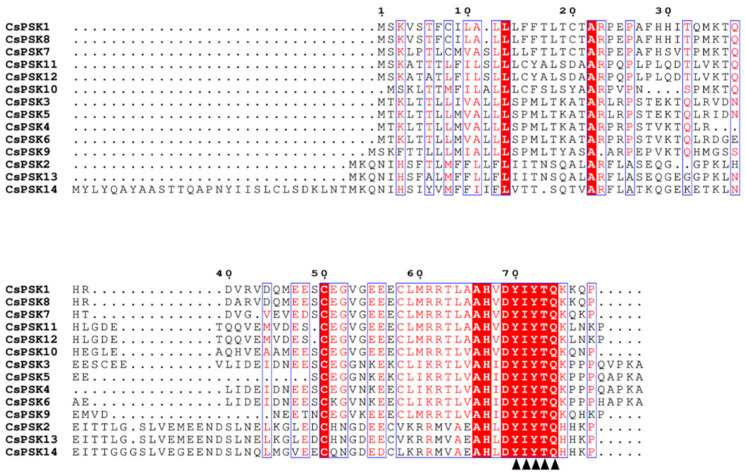
The multiple sequence alignment of the *CsPSK* gene family. Conserved pentapeptides are indicated by black triangles.

To further investigate the structure of the CsPSK genes, a multiple sequence alignment was performed on the CsPSKs. As shown in Figure 6, the CsPSKs contain the conserved functional domain of the PSK family, including the pentapeptide motif (YIYTQ).

### 2.4. Cis-Acting Element Analysis of CsPSKs Promoters

**Figure 7:**
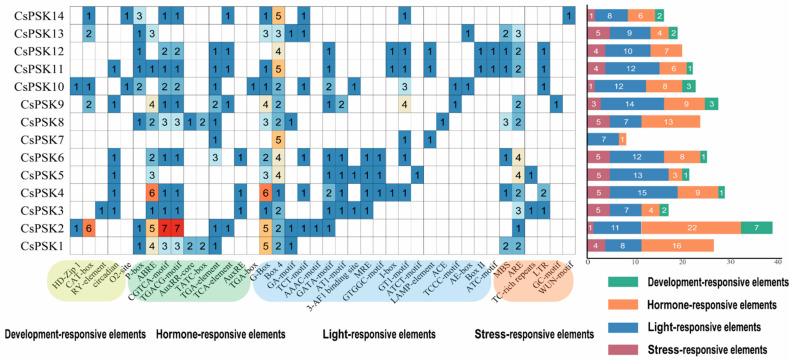
Analysis of cis-acting elements in the promoter regions of *CsPSK* genes. The numbers in the grid represent the quantity of cis-acting elements, while the color intensity indicates the abundance of these elements. The right side displays the statistics of cis-acting elements for each gene under four types, including light-responsive elements, hormone-responsive elements, stress-responsive elements, and development-related elements.

To further elucidate the potential physiological functions of the *CsPSK* gene family, we analyzed the 2kb region upstream of the transcription start site of the *CsPSK* genes. A total of 40 cis-regulatory elements were identified and categorized into four groups according to their functions, including light-responsive elements, hormone-responsive elements, stress-responsive elements, and development-related elements (Figure 7). Among the cis-acting elements of *CsPSK* genes, the light-responsive elements are the most abundant, while the development-related elements are the least frequent. Most *CsPSKs* contain four functional elements. Several cis-acting elements are specific to some *CsPSKs*, including RY-element (*CsPSK3*), TGA-box (*CsPSK10*), AAAC-motif (*CsPSK2*), ATCT-motif (*CsPSK5*), ACE (*CsPSK8*), GC-motif (*CsPSK9*) and WUN-motif (*CsPSK14*). All *CsPSKs* contain at least one hormone-responsive element, including methyl abscisic acid (ABA), auxin (IAA), Jasmonate (MeJA), gibberellin (GA) and so on. In addition, most *CsPSKs* contain stress-responsive elements, such as MBS (drought-inducibility) and ARE (anaerobic induction). Interestingly, we also identified cis-acting elements involved in circadian control in the *CsPSK* gene family.

### 2.5. Expression Profiling of CsPSKs Using RNAseq Data

**Figure 8:**
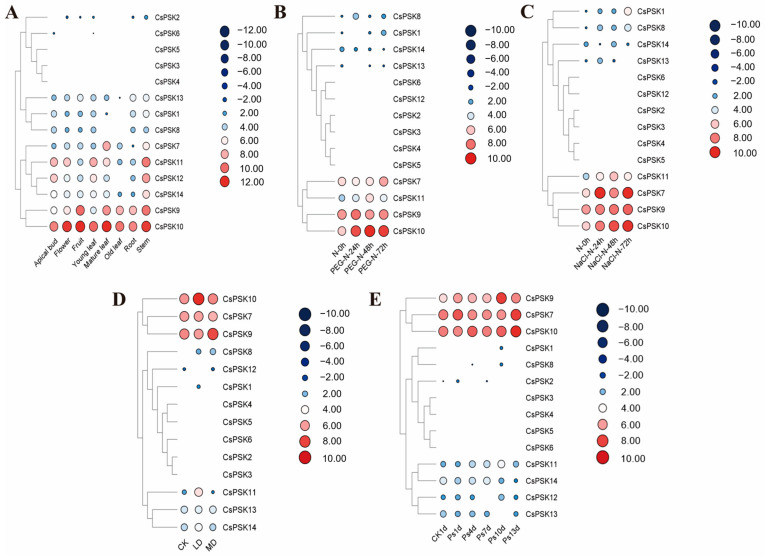
Expression patterns of *CsPSK* genes under different tissues and stress conditions. (**A**) Expression patterns of *CsPSK* genes in eight different tissues of tea plants. Expression responses of tea plants under (**B**) drought stress, (**C**) salt stress, (**D**) leafhopper infestation, and (**E**) gray blight infection. The size and color of the circles represent high and low expression levels, with red indicating high expression and dark blue indicating low expression.

The expression analysis of *CsPSKs* was investigated using publicly available transcriptome data from the TPIA. An analysis of *CsPSK* expression values (Transcripts Per Kilobase per Million mapped reads, TPM) revealed different expression patterns in eight tissues. The results showed that a majority of *CsPSKs* are expressed in most tissues, except *CsPSK3*, *CsPSK4*, and *CsPSK5* (Figure 8A). Two genes (*CsPSK9* and *CsPSK10*) are highly expressed in most tissues, suggesting their important roles in the development of tea plants. *CsPSK7* is mainly expressed in mature leaves and stems in tea plants, and *CsPSK14* has a specific high expression abundance in the stem.

To further investigate the stress response mechanism of *CsPSKs*, the expression of *CsPSKs* was analyzed by RNAseq data under different stress conditions. Abiotic stress, both drought and salt treatment, showed a similar expression trend, wherein *CsPSK1, CsPSK7, CsPSK8, CsPSK9, CsPSK10,* and *CsPSK11* were upregulated (Figure 8B,C). Additionally, under salt treatment (24h-NaCl), *CsPSK13* exhibited rapid increases in expression, while the expression of *CsPSK14* significantly decreased under drought and salt treatment. Figure 8D,E illustrates the expression pattern of *CsPSKs* under biotic stress. Under leafhopper infection, *CsPSK8*, *CsPSK9*, *CsPSK10*, and *CsPSK11* exhibited prominent expressions. The heatmap revealed an increased expression of *CsPSK7*, *CsPSK9*, *CsPSK10*, *CsPSK11*, *CsPSK12*, *CsPSK13*, and *CsPSK14* but a decreased expression of *CsPSK13* and *CsPSK14* in response to gray blight infection.

### 2.6. Expression Patterns of CsPSKs in Response to D. theae-sinensis

**Figure 9:**
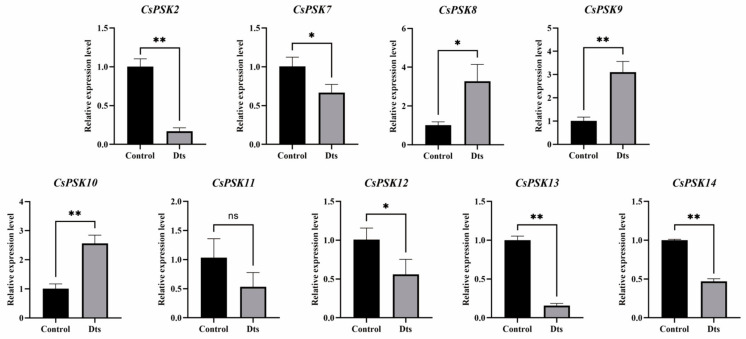
The relative expression patterns of *CsPSK* genes under *Discula theae-sinensis* infection within 12 h after inoculation. The error bars indicate the standard deviation (SD) based on three biological replicates. Asterisks (*) denote the level of statistical significance, where * indicates *p* < 0.05, ** indicates *p* < 0.01), and ns indicates non-significant. Dts, *D. theae-sinensis*.

To explore the response of *CsPSKs* to pathogen infection in tea plants, we conducted an RT-qPCR analysis of nine *CsPSKs* within 12 h after inoculation. As shown in Figure 9, different expression patterns of *CsPSKs* were observed under *D. theae-sinensis* infection. The expression levels of *CsPSK2*, *CsPSK7*, *CsPSK12*, *CsPSK13* and *CsPSK14* was significantly reduced when the tea plants were infected with *D. theae-sinensis*. Interestingly, three genes (*CsPSK8*, *CsPSK9* and *CsPSK10*) showed a notable upregulation in response to *D. theae-sinensis*, with expression abundance increasing 3–4 times, indicating their potential roles in the response of tea plants to *D. theae-sinensis*.

## 3. Materials and Methods

### 3.1. Identification and Characterization of CsPSK Genes

The PSK protein sequence of *A. thaliana* was downloaded from The Arabidopsis Information Resource (TAIR, https://www.arabidopsis.org, accessed on 3 August 2024). The *Camellia sinensis* genome and protein sequence were downloaded from the genome database Tea Plant Information Archive (TPIA, http://tpia.teaplants.cn/index.html, accessed on 3 August 2024). Using the AtPSK protein sequence as a reference, a comparative genome-wide search in the *C. sinensis* genome database was conducted using the blast program of TBtools-II v2.142 software (E-value < 1 × 10^−2^) [26]. Subsequently, all predicted CsPSK proteins were checked and subjected to NCBI-CDD (https://www.ncbi.nlm.nih.gov/Structure/bwrpsb/bwrpsb.cgi, accessed on 3 August 2024) and MEME (https://meme-suite.org/meme/, accessed on 3 August 2024) analysis to confirm conserved motifs and domains. The physicochemical properties of the CsPSK proteins were analyzed using Protparam (https://web.expasy.org/protparam/, accessed on 3 August 2024). Subcellular localization was predicted using Cell-PLoc 2.0 (http://www.csbio.sjtu.edu.cn/bioinf/Cell-PLoc-2/, accessed on 10 February 2025). Furthermore, the chromosome location of *CsPSKs* was conducted with TBtools-II v2.142 software, and the genes were named based on genomic sequence ID.

### 3.2. Phylogenetic and Genome Synteny Analysis of CsPSK Genes

The PSK protein sequences of *C. sinensis*, *A. thaliana*, *G. max*, *L. japonicus*, *M. truncatula*, *Zea mays*, and *Solanum lycopersicum* were subject to multiple sequence alignment using ClustalW program in MEGA 11 software (Table S1); the phylogenetic tree was constructed using a neighbor-joining (NJ) method with bootstrap 1000, a JTT model and pairwise deletion [27]. Then, the tree was beautified using the online tool Evolview [28]. Genome synteny analysis, non-synonymous nucleotide substitution rates (Ka), synonymous nucleotide substitution rates (Ks), and the ratio of Ka/Ks were conducted using Tbtools-II software.

### 3.3. Sequence Alignment and Gene Structure Analysis of CsPSK Genes

The amino acid sequences of CsPSK were aligned using ClustalW, and the results were visualized with ESPript 3.0 (https://espript.ibcp.fr/ESPript/cgi-bin/ESPript.cgi, accessed on 6 August 2024). The coding sequences (CDSs) and Untranslated Regions (UTRs) of the *CsPSK* genes were extracted based on the genome annotation information of *C. sinensis*. TBtools-II v2.142 was used to display the phylogenetic trees, motif patterns, domain patterns, and gene structures.

### 3.4. Identification and Analysis of CsPSK Genes Cis-Acting Elements

The promoter regions (2 kb upstream) of the *CsPSK* genes coding regions were identified through searches of the *C. sinensis* genome database. The promoter sequences were then submitted to the PlantCARE website (https://bioinformatics.psb.ugent.be/webtools/plantcare/html, accessed on 10 August 2024) for further analysis of cis-acting elements [29].

### 3.5. Analysis of CsPSK Genes Expression Pattern Using RNAseq Data

Transcriptome sequencing data of tea plants were downloaded from the tea plant genome database website TPIA. The expression abundance of *CsPSK* genes in different tissues, abiotic stress and biotic stress was represented using Transcripts Per Million (TPM) values.

### 3.6. Plant Materials and Pathogens

The tea cultivar ‘Longjing 43’ (LJ43), widely cultivated in China, was utilized in this study. The identification of pathogenic fungi was identified using an approach similar to that described in previous studies [19,20,30], confirming the fungus as *Discula theae-sinensis* (Figure S1). Leaf samples of LJ43 were collected 12 h after inoculation with *D. theae-sinensis*, with three biological replicates being obtained. Samples were immediately placed in liquid nitrogen after collection for subsequent experiments.

### 3.7. RNA Extraction, cDNA Synthesis and RT-qPCR Expression Analyses

Using the RNA Simple Total RNA Kit (TIANGEN, Beijing, China), RNA was extracted. RNA qualities were determined, and RNA concentrations were standardized for consistency. The cDNA was generated using a reverse transcription Kit (AG, Hunan, China). An RT-qPCR analysis was performed using a SYBR Green Pro Taq HS fluorescent dye kit (AG, Hunan, China). The gene-specific primers for *CsPSKs* used in RT-qPCR were designed using the primer3plus online website (https://www.primer3plus.com/, accessed on 15 August 2024) (Table S5). The *CsGAPDH* was employed to normalize the target gene and correct for sample-to-sample variation [31]. The reaction procedure is as follows: 95 °C for 30 s, followed by 45 cycles of 95 °C for 10 s and 60 °C for 30 s. Then, fluorescence was measured using a 60–95 °C melting curve to detect a specific peak in each gene. The gene expression levels were quantified relative to the control using the 2^−∆∆CT^ method. [32]. Three biological replicates were performed. Statistical significance was analyzed using SPSS version 25 (IBM Corporation, Armonk, NY, USA), and the difference between the two samples was determined by an independent samples *t*-test. Graphs were generated using GraphPad Prism 9.5.0 (GraphPad Software, San Diego, CA, USA).

## 4. Discussion

The *CsPSK* gene family encodes a class of sulfated peptides predominantly present in plants and is involved in regulating diverse developmental pathways and stress responses. The PSK genes have been systematically studied in many plants [7], but not in tea plants. Therefore, we conducted a comprehensive analysis of the *CsPSK* gene family in the tea plant to determine the characteristics of *CsPSK* genes.

Here, we surveyed the whole tea plant genome using various bioinformatics methods and obtained 14 members of the *CsPSK* gene family. The 14 PSK genes of *C. sinensis* are distributed across seven tea plant chromosomes, with several *CsPSK* genes forming obvious gene clusters (Figure 1). These gene clusters demonstrate high homology and are situated within the same branch of the phylogenetic tree (Figure 2). Notably, certain genes located on different chromosomes are also grouped with these gene clusters (such as *CsPSK2*, *CsPSK13* and *CsPSK14*; *CsPSK3*, *CsPSK4*, *CsPSK5*, *CsPSK6* and *CsPSK9*; *CsPSK11*, *CsPSK12* and *CsPSK10*), which may suggest an evolutionary adaptation in tea plants to enhance their resilience to environmental changes. The mapping of the *CsPSK1* gene to Contig950 may be due to the limited depth of genome sequencing. Furthermore, the variation in exon–intron structures is closely linked to gene duplication and functional diversity [33]. Interestingly, *CsPSK10*, *CsPSK11*, and *CsPSK12* are evolutionary homologous genes, but *CsPSK10* is located on a different chromosome from *CsPSK11* and *CsPSK12* (Figure 3). Notably, no PSK genes exhibiting collinearity with *A. thaliana* and *S. lycopersicum* were detected on chromosomes 3 of *C. sinensis*, suggesting that these genes may possess unique functions in tea plants (Figure 4). All the *CsPSK* gene family members contain the PSK superfamily’s conserved domains and pentapeptide motifs (Figure 5 and Figure 6). A comprehensive analysis of *CsPSK* gene structures revealed that the *CsPSK7* is characterized by the absence of motif 3 and consists of five exons (Figure 5), differing from *CsPSK1* and *CsPSK8*, which are located within the same phylogenetic branch. Genes that lack introns can rapidly respond to changes in environmental factors and are primarily induced by stress [34]. Eight *CsPSK*s genes are devoid of introns, and they exhibit a similar pattern of distribution (Figure 5). Furthermore, the number of *CsPSK* genes is significantly higher compared to other species, such as model plant species tomato [10] and Arabidopsis [5]. An interspecific synteny analysis among *C. sinensis*, *A. thaliana*, and *S. lycopersicum* further elucidated the homologous relationship within the PSK gene families. According to expression analysis, *CsPSK10* and *CsPSK11*/*CsPSK12* displayed distinct expression patterns, a similar trend observed between *CsPSK7* and *CsPSK8* to disease infection (Figure 9). These results suggest that the *CsPSK* gene family in tea plants may have undergone functional divergence during evolution, likely in response to selective pressures from the external environment. Furthermore, *CsPSK2*, *CsPSK13*, and *CsPSK14* are grouped within the same clade, with their expression levels downregulated upon infection by *D. theae-sinensis* (Figure 9), while *CsPSK2* and *CsPSK13*, which are part of the same gene cluster, suggest potential functional redundancy or co-regulation. These results indicate that the *CsPSK* gene family in *C. sinensis* has diverged throughout evolution.

Genes within the same clade of the phylogenetic tree may encode sequences with similar biological functions [35]. We constructed the phylogenetic tree, including the PSK protein sequence of monocot and dicot plants (Figure 2). The result showed that all maize PSK sequences were grouped within the same clade, likely due to maize being the only monocot species represented in the phylogenetic tree. Additionally, phylogenetic analysis revealed that the PSK protein sequences in tea plants belong to the PSK-α clade, which is consistent with their pentapeptide motif. Interestingly, many proteins within the same clade as tea plant PSKs have been found to play crucial roles in regulating pathogen stress and promoting tissue growth. For instance, *AtPSK4* has been implicated in the regulation of root and leaf growth [36], the induction of male sterility [37], and the regulation of organisms’ immune responses to pathogens [12,13], primarily based on overexpression studies. Similarly, *AtPSK5* functions as a crucial signaling molecule regulated by *ERF115*, which controls the division of quiescent center cells and the replenishment of stem cells, thereby influencing root development and stress responses in plants, as demonstrated through overexpression experiments [38]. In tomato, *SolyPSK3* and *SolyPSK3L* enhance immunity against *B. cinerea* based on the VIGS method [10], while *SolyPSK1* and *SolyPSK6* promote drought-induced flower abscission [39]. The *CsPSK* genes in tea plants clustered within the same branch as some of the genes mentioned above, suggesting homology and potential functional similarities (Figure 2). Furthermore, plant peptides can influence hormone signaling pathways, such as auxin and SA, thereby balancing plant growth and immune responses [40]. For example, in *A. thaliana*, PSKR1 can suppress the SA signaling pathway to limit the response to *Pseudomonas fluorescens* while simultaneously enhancing photosynthesis to promote growth [23]. The PSK signaling in tomato increases cytosolic Ca^2+^ concentration by enhancing the binding activity between calmodulin and the auxin biosynthetic proteins YUCs, thereby promoting auxin-dependent immune responses [10]. In our study, the *CsPSK* gene family is associated with numerous cis-acting elements, including those responsive to ABA, IAA, GA, and SA, indicating that the *CsPSK* gene family may enhance the ability to respond to stress conditions for tea plants.

Gene expression analysis provides valuable insights into gene distribution and function, helping us to better understand their potential roles in various biological processes [41,42]. In Arabidopsis, *PSK1* exhibited expression across all cell layers, with stronger expression in the epidermis, while *PSK2*, *PSK3*, *PSK4*, and *PSK5* were primarily detected in the central cylinder, indicating their potential key roles in root growth and development [43]. The analysis of the tissue-specific expression patterns of the genes provides important insights into their functional roles in plants [44]. Our results showed that *CsPSKs* are expressed in various tissues (Figure 8). For instance, *CsPSK7*, *CsPSK9*, and *CsPSK10* are predominantly expressed in mature leaves and show strong responses to gray blight infection (Figure 8). At the transcriptional level, the expression abundance of four tomato PSK genes (*SolyPSK3*, *SolyPSK3L*, *SolyPSK4* and *SolyPSK7*) was significantly influenced by *B. cinerea* infection [10]. Subsequent validation through VIGS revealed that silencing *SolyPSK3* and *SolyPSK3L* significantly increased tomato susceptibility to *B. cinerea* [10]. In our study, RT-qPCR results showed that the expression level of *CsPSK10*, *CsPSK8*, and *CsPSK9* significantly increased in response to *D. theae-sinensis* infection within 12 h after inoculation (Figure 9), suggesting their potential involvement in the regulation of tea plant disease resistance. These findings provide novel insights into the evolutionary relationships and potential functions of the *CsPSK* gene family, which may be useful for further research on the mechanisms of peptide signaling-mediated disease resistance in tea plants.

## 5. Conclusions

In this study, we performed a comprehensive genome-wide analysis of the *CsPSK* gene family in *C. sinensis*. We identified a total of 14 *CsPSK* genes, and all these *CsPSKs* belong to the PSK-α. We then analyzed their physicochemical properties, phylogenetic relationships, gene duplication events, gene structures, cis-acting elements, and expression patterns. Additionally, we examined the response of *CsPSK* genes to *D. theae-sinensis* infection within 12 h after inoculation, with eight genes showing significant differential expression following pathogen infection. Overall, the evolutionary and expression pattern analyses provide valuable insights into the role of *CsPSKs* in plant stress responses and offer new directions for future research on their functions and molecular mechanisms.

## Figures and Tables

**Table 1:** Identification information of CsPSKs in *C. sinensis*.

Gene ID	Gene Name	Number of Amino Acid	Molecular Weight (Da)	Theoretical pI	Hydropathicity	Instability Index	Aliphatic Index	Predicted Subcellular Location
CSS0000381.1	CsPSK1	78	9060.51	6.39	−0.265	66.1	75	Nucleus
CSS0006810.1	CsPSK2	93	10,688.20	5.38	−0.3	51.7	90.22	Nucleus
CSS0021707.1	CsPSK3	89	10,024.65	5.39	−0.348	61.95	100.79	Chloroplast. Nucleus.
CSS0026516.1	CsPSK4	79	8877.49	8.66	−0.225	63.08	101.27	Nucleus
CSS0031911.1	CsPSK5	75	8409.97	9.51	−0.352	57.61	93.73	Chloroplast. Nucleus.
CSS0036107.1	CsPSK6	84	9387.01	7.79	−0.321	55.72	96.43	Nucleus
CSS0039448.1	CsPSK7	78	8698.11	5.47	−0.067	56.7	82.44	Chloroplast
CSS0040959.1	CsPSK8	78	9001.44	6.39	−0.272	66.1	72.56	Nucleus
CSS0047712.1	CsPSK9	77	8800.24	5.85	−0.291	67.2	82.34	Nucleus
CSS0048145.1	CsPSK10	78	8788.15	5.2	−0.133	60.24	82.56	Chloroplast. Nucleus.
CSS0048277.1	CsPSK11	83	9311.70	4.77	−0.194	53.44	95.18	Chloroplast
CSS0049317.1	CsPSK12	83	9281.67	4.77	−0.164	53.44	96.39	Chloroplast
CSS0049961.1	CsPSK13	95	10,821.30	5.06	−0.312	50.03	89.37	Nucleus
CSS0050359.1	CsPSK14	124	14,060.96	5.26	−0.303	41.29	83.39	Nucleus

## Data Availability

The data presented in this study are available on request from the corresponding author. The data are not publicly available due to privacy.

## Supplementary Materials

The following supporting information can be downloaded at: https://www.mdpi.com/article/10.3390/ijms26062418/s1.
