# Supplementary figures and images for: Genome-Wide Identification and Expression Analysis of Phytosulfokine Peptide Hormone Genes in Camellia sinensis
Source: Int J Mol Sci. 2025 Mar 7;26(6):2418. doi: 10.3390/ijms26062418 (PMC11942274; doi:10.3390/ijms26062418)

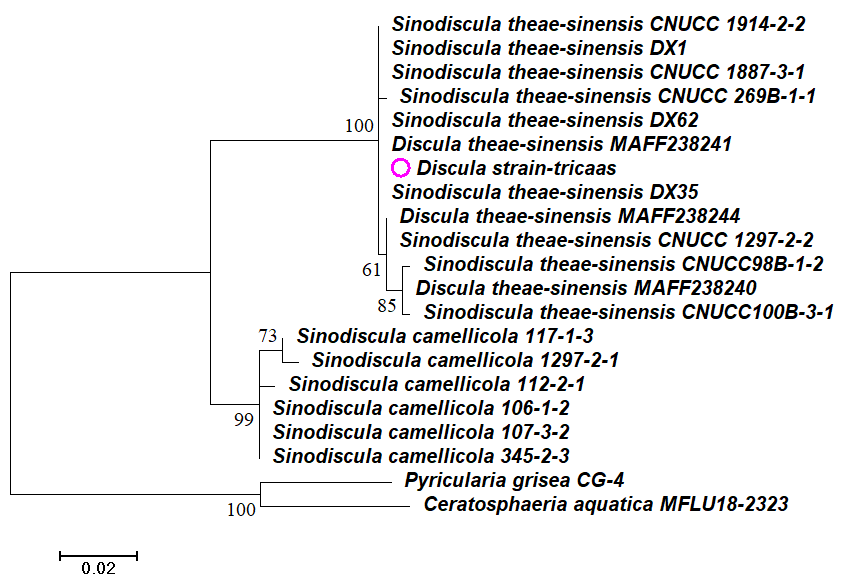

Supplement: Supplementary file 1 [file ijms-26-02418-s001.zip › ijms-3463928 -Fig. S1.tif]
